# Diet Impact on Obesity beyond Calories and Trefoil Factor Family 2 (TFF2) as an Illustration: Metabolic Implications and Potential Applications

**DOI:** 10.3390/biom11121830

**Published:** 2021-12-04

**Authors:** Abdelaziz Ghanemi, Mayumi Yoshioka, Jonny St-Amand

**Affiliations:** 1 Functional Genomics Laboratory, Endocrinology and Nephrology Axis, CHU de Québec-Université Laval Research Center, Québec, QC G1V 4G2, Canada; abdelaziz.ghanemi@crchudequebec.ulaval.ca (A.G.); mayumi.yoshioka@crchudequebec.ulaval.ca (M.Y.); 2 Department of Molecular Medicine, Faculty of Medicine, Laval University, Québec, QC G1V 4G2, Canada

**Keywords:** obesity, diet, calories, energy metabolism, trefoil factor family member 2, high-fat diet

## Abstract

Obesity is a health problem with increasing impacts on public health, economy and even social life. In order to reestablish the energy balance, obesity management focuses mainly on two pillars; exercise and diet. Beyond the contribution to the caloric intake, the diet nutrients and composition govern a variety of properties. This includes the energy balance-independent properties and the indirect metabolic effects. Whereas the energy balance-independent properties are close to “pharmacological” effects and include effects such as antioxidant and anti-inflammatory, the indirect metabolic effects represent the contribution a diet can have on energy metabolism beyond the caloric contribution itself, which include the food intake control and metabolic changes. As an illustration, we also described the metabolic implication and hypothetical pathways of the high-fat diet-induced gene Trefoil Factor Family 2. The properties the diet has can have a variety of applications mainly in pharmacology and nutrition and further explore the “pharmacologically” active food towards potential therapeutic applications.

## 1. Obesity and the Ongoing Coronavirus Disease 2019 Crisis

Obesity, as an epidemic health problem [1], represents a topic of a continuously increasing number of studies surrounding different fields, ranging from obesity development and pathogenesis to the related diseases and health problems, at different life stages and physiological or pathological statuses. The basic definition of obesity is a lost balance between energy intake and energy expenditure that leads to an accumulation of excessive calories, mainly as lipid storage within the adipocytes in a distribution pattern that depends on genetic and environmental factors [2,3]. Such energy imbalance results from the superiority of the energy intake represented by the food intake compared to the energy expenditure represented mainly by the basal metabolic rate [4], physical activity/exercise [5,6] and thermogenesis [7,8,9]. Obesity can also develop as results of gene disruption or deficiency, such as the Brd2 gene [10,11] and the leptin gene [12,13], both leading to metabolic pathways towards an obesity-related phenotype. In addition, obesity has also epigenetics factors [14,15,16,17]. The related adiposity distribution depends on many factors and also impacts the pathological outcomes of obesity [18]. The pathogenesis of obesity has been discussed from a variety of perspectives, including broken energy homeostasis [18], microbiota contribution [19], neuroendocrine reprogramming [20] and even toward considering obesity as a disease [2,21]. The complexity of the molecular and cellular mechanisms governing obesity establishment, development and pathogenesis are poorly understood, which limits its research advances and therefore the development of efficient molecular therapies. The danger of obesity and increased adiposity results from their various impacts on health in terms of diseases risks and disturbed biological functions [22]. That includes metabolic disorders [23], cardiovascular diseases [24,25], dyslipidemia [26], impaired regeneration [27], inflammation [28], kidney disease [29], cancer [30], diabetes [24], impacts on immunity and antibodies [31,32,33,34], age-related cognitive decline [35], respiratory complications [36,37] and infertility [38,39].

It is worth noting that the ongoing coronavirus disease 2019 (COVID-19) crisis has increased the importance of obesity research because of both the impact of the COVID-19 pandemic (and the related measures [40,41]) on obesity development [42] as well as the obesity-induced increased vulnerability to COVID-19 [43,44,45]. The measures imposed by the governments and health authorities, including confinement and its consequences (sedentary lifestyle, reduced physical activity, increased food intake, disturbed sleeping, etc. [41,42,46]) are towards worsening obesity rates worldwide. This includes the greater stress that was related to a higher body mass index, an increased working time, a higher anxiety level and less time to spend on weight management efforts [47] during this COVID-19 crisis. On the other hand, obesity consequences on the prognosis of patients with COVID-19 include increased hospitalization, intensive care unit admission and mortality [48]. The mechanisms beyond such increased vulnerability are diverse and include the chronic inflammatory character of obesity [49], immune dysregulation [50], excessive oxidative stress [51] and probably metabolic dysfunction and endothelium imbalance as well [52]. Furthermore, in addition to the direct impact of obesity on COVID-19 severity, obesity would also have indirect impacts on COVID-19. Obesity-related diseases and health conditions represent risk factors toward increasing the vulnerability to COVID-19. Diabetes, one of the most known diseases associated with obesity [53,54], represents a risk factor for severe COVID-19 [55,56,57]. Cardiovascular diseases, which have their risks increased in obese patients [25,58,59], also represent risk factors for COVID-19 [60]. Other obesity-related health problems, such as hypertension and kidney and liver diseases [57], do represent risk factor for the severe forms of COVID-19. In addition, studies have also explored the fact that COVID-19 could also worsen or lead to other health problems, including diabetes [56,61], and possibly lead to a post-COVID-19 multi-level health crisis [62]. That would lead to a vicious cycle involving obesity, diabetes and COVID-19, among other pathologies that also increase COVID-19 severity. Moreover, the impacts of obesity on immunity [31,32,33,34] threaten to reduce the efficacy of anti-COVID-19 vaccines [63] that represent the best hope for humans to see an end of this pandemic.

The heavy consequences of obesity on health as well as on economy [64] and society [65,66] have led to the development of various pharmacotherapies [67] for obesity and the necessity to have other medications in development [68]. However, the main approaches to manage obesity remain exercise [69,70] and diet [71]. Along with the energy expenditure (exercise [6], thermogenesis [72], etc.), the diet represents the energy intake part of the caloric balance. Therefore, it is important to describe the nutritional aspects of the diet. Thus, the main focus of the nutritional research in obesity emphasizes the caloric density and the need to limit the caloric intake [73] to create a caloric deficiency leading to adiposity reduction and weight loss. Within this context, high-fat diet (HFD) and high-sugar diet have a high importance. HFD diet is characterized by both its high caloric density and a limited satiety. In addition, sugar consumption has even been described in the context of addiction [74] that contributes to obesity development [75]. Furthermore, the combined effect of high fat and high sugar has the most deleterious effects, as suggested by the work of Ghosh et.al. [76]. This gives both HFD and high-sugar diet a special status within nutrition research in the context of obesity and weight management.

## 2. Diet and Calories-Independent Patterns

**Figure 1:**
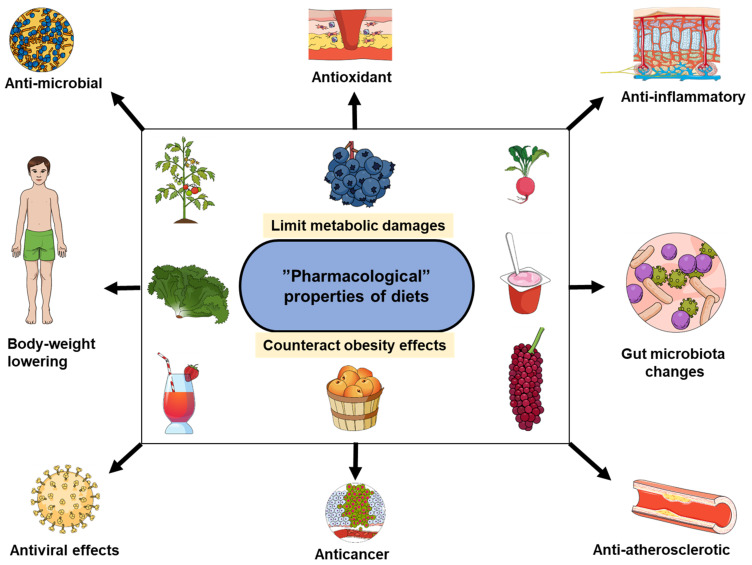
The energy balance-independent diet benefits are close to pharmacological effects. The diverse benefits related to food include properties that counteract consequences of obesity, such as inflammation, cancer risk and metabolic damages.

The important diet caloric contribution to obesity is well documented, and various diets have been studied and compared in the context of prevention and treatment of obesity [77,78,79,80,81,82,83]. The research “obesity-diet” mainly focuses on controlling/limiting the caloric intake to manage obesity or reduce the adiposity. We describe fat and sugar diets as key contributors to the caloric intake. However, sugar and lipids cannot be seen only as caloric resources or “fuel”; they also play important biological roles within the cellular and subcellular structures, biochemical pathways, heat insulation, etc. Similarly, and in addition to the important direct impacts diet has on obesity, the in vivo diet consequences include two main patterns, energy balance-independent and those indirectly impacting the energy metabolism. In these two, the diet not only directly impacts the energy balance by increasing the caloric intake. The energy balance-independent diet benefits are close to “pharmacological” effects in which the diet elements can be described as functional foods or nutraceuticals [84,85,86,87,88,89]. Indeed, diet contents have been reported with a variety of properties that can be pharmacologically explored, including antioxidant pattern [90,91], anti-inflammatory [92,93], anti-microbial, anti-fungal, anti-diabetic and anti-atherosclerotic activity [94], anticancer properties [95,96], antiviral effects [97], body weight lowering fibers [98], protection against endogenous exposure to persistent organic pollutants [99] and gut microbiota changes [99]. In addition, consumption of elements, such as calcium (calories-free), has also been shown to impact obesity development [100,101]. Furthermore, some compounds from vegetables and fruits can be converted to hormone-like active substances with biological actions [102]. All these elements illustrate how the diet impacts on obesity go beyond the caloric density (quantity) but also include the diet composition (quality) as exemplified in Figure 1. Thus, a balanced diet within an anti-obesity approach should not be limited to a caloric intake control but needs also to make use of such “pharmacological” properties to both optimize the anti-obesity effects and also obtain effects such as anti-inflammation that will counteract outcomes seen during obesity (inflammation, increased risk cancer, etc.). Within this context, some concepts reported in the literature, such as “eat to treat” [103] would find there explanatory mechanisms and underlying pathways in those calorie-independent impacts of diet. Some of these properties (potential antimicrobial, antioxidant and anti-inflammatory) have also led researchers to suggest the Mediterranean diet as a nutritional approach for COVID-19 [104].

**Figure 2:**
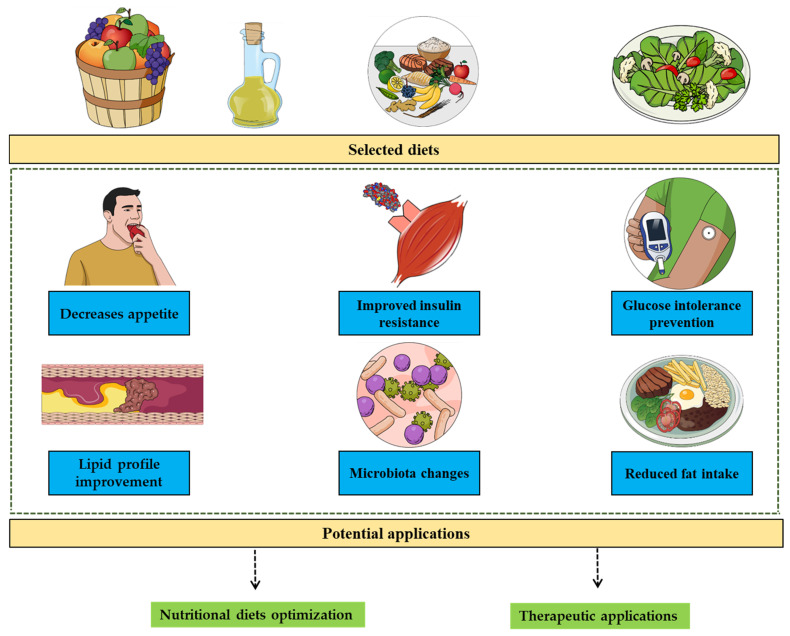
Indirect impacts diet has on energy metabolism. Some diets have the ability to impact the energy metabolism through insulin sensitivity improvement, appetite control, microbiota changes-related metabolic changes and improving the lipids profile. These properties are independent from the caloric intake that comes with such diets due to caloric independent-patterns.

On the other hand, the second perspective is that dietary elements can indirectly impact the energy metabolism. Among the good examples are: (i) the red pepper decreases appetite [105], reduces fat intake [106] and increases satiety [107], (ii) oligofructose reduces hunger [108], (iii) dietary fiber potentiates weight regulation [109] and (iv) fenugreek fiber reduces energy intake and increases satiety [110]. In addition, the increased insulin resistance following the ingestion of a protein source mirroring western diet (compared to provision of casein) [111], prevention of obesity-related glucose intolerance by the salmon peptide fraction [112], the benefits of fish oil on diet-induced insulin resistance [113], the fish oil diet induced both reduction in body weight gain in ob/ob mice [114] and improvement of glucose intolerance in HFD-fed mice [115], the role of n-3 polyunsaturated fatty acid in insulin resistance prevention [116] that involve controlling adipose tissue inflammation in its mechanism [117], HFD effects on gut hormones production [118], the HFD-induced reduction of the sensitivity to satiety signals [119], blood lipid profile and body fat distribution improvement with fish oil [120], and appetite control [121] and food intake modification [122] of coffee/caffeine are also important illustrative properties mediated by diet and represent an indirect energy balance management. All these paths represent illustrations of how selecting diet can impact the energy balance and the metabolism beyond the simple caloric intake related to the diet (Figure 2). These properties can be explored to optimize nutritional approaches and therapeutic development. This field is well supported by functional genomics studies exploring the transcriptomic changes depending on the diet [123] which highlight the links between the regulated genes and the related underlying metabolic pathways.

Moreover, the key consequences of the diet on microbiota composition, which has a significant metabolic importance, reflects another indirect impact diet has on energy balance. Indeed, some diets rich in or with a supplementation of probiotics, prebiotics or synbiotics can modulate the microbiota [124]. For instance, a pro-inflammatory gut microbiota development is promoted by HFD [125], and no microbiota composition pattern was associated with vegan or vegetarian diets [126] which reflects some microbiota effects. The microbiota changes result in adaptations, including metabolic improvement [127,128] and even improving/treating obesity [19,124,129]. Importantly, the fact that different diets lead to different changes in gut microbiota composition [130] and that different diets also lead to different transcriptome expression [123] both suggest a diet-specific metabolic phenotype. Beyond the aspects of potential therapeutic effect and the impacts a diet has on biological processes, including genes and metabolic pathways represents a growing area that could lead to therapeutic applications.

In addition to obesity, ageing also represents an important risk factor for a variety of diseases and health problems including cardiovascular disease [131], loss of neuroplasticity [132], cerebral ischemia [133], osteoporosis [134], pulmonary disease, cancer [135] and neurodegenerative disease [136]. Therefore, healthy ageing will contribute to reducing the risk and the severity of various diseases. For that, a healthy lifestyle that includes, in addition to exercise [137,138,139], sleep quality [140] and metal health [141], optimized diets choice is a key to achieve healthy ageing. Within this context, and based on the ageing-related pathways, mainly the oxidative stress [142] and redox regulation [143] involving reactive oxygen and nitrogen species [135] and free radicals [144,145], a diet of selected properties would contribute to healthy ageing. Indeed, food and beverages with properties ranging from antioxidant [146] and anti-inflammatory to anticancer and metabolic benefits will reduce the risks associated with ageing. For instance, diet choice toward white meat and fish can reduce the risk of age-related cognitive decline [147], and dietary antioxidants contribute to healthy ageing [148]. Such impacts of diet management in ageing [146] will lead to further development of geriatrics and elderly care beyond pharmacology.

## 3. Diet and Trefoil Factor Family: From Gastrointestinal Protection to Metabolic Implications

Following the same line of thought highlighting the indirect impacts of the diet, we provide an illustrative example of the indirect impacts of diet on energy balance mediated by trefoil factor family (TFF). Indeed, the indirect effect on metabolism can also be illustrated by the diet consequences on selected metabolic patterns related to the energy balance through the TFF. The TFF is a family of secreted protease-resistant peptides [149] with intramolecular disulfide bonds [150], trefoil domain(s) [151] and C-terminal dimerization domain, and which have important roles in mucosal protection and post-injury mucosal repair [152], epithelial migration promotion [153] and mucosal healing [154]. Whereas *Tff1*, *Tff2* or *Tff3* knock-out (KO) in mice led to gastrointestinal impairment [155], TFF have a therapeutic potential to treat gastrointestinal disorders [156]. While TFF1, or pS2, is expressed mainly in the stomach [152] without a known receptor (although a basolateral binding site in the gastrointestinal epithelial was suggested for the three TFFs [150]) or detailed metabolic implications, TFF3, or ITF, expressed in both the small (duodenum, jejunum and ileum) and large intestine [152] and in other tissues, such as the liver [157] and the nervous system [158], is still without a validated receptor [159,160], but its expression is regulated by food intake [161]. The metabolic implications of TFF3, although not well known, are also worth exploring as it has been shown to regulate the glucose metabolism in the liver [157], and its deficiency leads to an affected hepatic lipid metabolism but with an improved glucose utilization [162]. On the other hand, TFF3 improves HFD-induced hepatic steatosis [163] and, in a diet-induced obesity mouse model, TFF3 improved glucose tolerance [161].

On the other hand, TFF2, known as spasmolytic peptide (SP), is a very stable small protein [164] expressed in the duodenum [152], the stomach, the colon, immune organs, leucocytes [164], macrophages, spleen cells [165], lymphocytes [165] hypothalamus [166] and the kidneys [167]. Unlike TFF1 and TFF3, it has been further studied in diverse context, including the energy metabolism. Indeed, TFF2 has been shown/suggested to play roles in anti-inflammatory pathways [165,168], counteracting the immune-mediated damage resulting from the HFD [169] and immune response [165,170]. Among the TFFs, TFF2 has been shown to play a role in obesity and metabolic disorders, mainly after its characterization as an HFD-induced gene and the work that explored its metabolic implications. Briefly, functional genomics studies characterized *Tff2* as an HFD-induced gene [171,172]. Thus, TFF2/*Tff2* expression has been suggested as an indicator of the severity of the HFD-induced obesity with a variety of potential applications [173]. Importantly, *Tff2* KO protected the mice from the HFD-induced obesity [166], which was explained by a metabolic phenotype toward an increased energy expenditure [174]. As an attempt to explain all these findings, we have hypothesized that the HFD-induced overexpression of TFF2 would represent a mechanism of adaptation to the HFD ingestion leading to an increased lipid absorption and storage via TFF2-stimulated receptors, as we previously reviewed [175]. This correlates with the exacerbated weight loss resulting from TFF2 deficiency [164].

Regarding the molecular and cellular pathways beyond such TFF2 metabolic roles, we highlight the receptor chemokine (C-X-C motif) receptor 4 (CXCR4), that belongs to the important family of G protein-coupled receptor [176,177], which is a known TFF2 receptor, including in cancer cell lines [178]. For instance, during development, the embryonic insulin-producing cells are maintained by the TFF2/CXCR4 axis [179]. This could indicate an insulin-mediated metabolic implication of TFF2, especially that cell proliferation in pancreatic β-cells is promoted by TFF2 through CXCR-4-mediated phosphorylation [180], and the *Tff2* KO reduced insulin serum levels in mice [166]. Furthermore, the expression of CXCR4 in key metabolic tissues, such as the liver [181], adipose tissue [182] and muscles [183] as well as the central nervous system [184] and the digestive tract [185], correlates with the metabolic changes observed in *Tff2* KO mice. Moreover, the increased expression of CXCR4 in situations in which TFF2 is also overexpressed, such as cancer [181,186], further supports such molecular links. Importantly, the CXCR4-defeciency in adipocytes leads to exacerbated obesity and impairs the brown adipose tissue thermoregulatory process [182]. This correlated with our previous hypothesis in which TFF2 would be a signal aiming to limit the food intake and obesity development since CXCR4 deficiency would prevent TFF2 from playing the related metabolic roles in limiting obesity and thus, explains the exacerbated obesity.

In addition, the fact that *Tff2* KO mice have an increased expression of agouti-related protein (AgRP) in the arcuate nucleus of the hypothalamus [166] may suggest that the HFD-induction of *Tff2* [171,172] could limit the expression (or at least prevent the increased expression seen in *Tff2* KO mice) of AgRP and thus limit its orexigenic and energy expenditure reduction effects [187], thus supporting the hypothesis that TFF2 could be a lipid-induced signal aiming to shift the energy balance towards counteracting the HFD-related excessive caloric intake. On the other hand, the melanocortin system, involved in the control of energy balance [188,189,190], receptors have AgRP as their antagonists [191]. Thus, suggesting a possible molecular link between TFF2 and the melanocortin system via the AgRP. The underlying molecular pathways are yet to be elucidated, and other works pointed out the need to clarify the roles of miRNAs in the TFF2-related processes [149,155].

The interesting part of TFF is that they are mainly expressed in the gastrointestinal system [152,192,193,194] which is the location in which the nutrients are fully digested into their basic forms to be absorbed [195,196]. Thus, there would be potential pathways according to which there is a detection of those nutrients [197] in the gastrointestinal system resulting in regulatory signals sent to the energy metabolism tissues (both regulatory as well as the key metabolic tissues) to trigger biological processes toward a metabolic adaptation [198] to such energy intake depending on the detected nutrients. This explains that stomach and intestine represents the starting point of energy balance regulatory signals as candidates for pharmacological targeting of TFF. Regarding TFF2 perspectives, the ultimate goal would be to identify a targetable lipid-specific signaling pathway(s) in order to control the food intake as well as other metabolic patterns. Unlike glucoses, for which we have the insulin as an induced acute regulatory metabolic signal, we are yet to identify a lipid-specifically induced acute signals equivalent to insulin. Identifying such signal(s) would allow researchers to target the food intake and control the appetite. The rationale beyond the potential targeting of TFF2 is that *Tff2* expression is induced by HFD within 30 min from the meal intake, a perfect therapeutic timing. Although we have the leptin concertation that changes with adiposity/obesity development [199,200,201] as well as fasting and re-feeding [202,203,204], identifying acute appetite/metabolic signals specific for lipids would still represent a key breakthrough with various therapeutic and mechanistic potential applications and implications.

## 4. Perspectives

The traditional approach of measuring the impact of diet on energy metabolism and obesity was limited to the mathematical evaluation of the caloric intake as compared to the energy expenditure. Now, the new advances on food chemistry and the related biological impacts allow researchers to expand the approach of diet impacts on obesity development beyond the direct caloric input. The diet can directly influence the energy balance toward certain metabolic phenotypes or can induce signals, such as TFF2, that would modify metabolic pathways (indirect impact of the diet). These concepts related to the non-caloric patterns of the diet would allow a deeper exploration of how diet choices affect the energy balance. Therefore, allowing an optimization of the diet in order to target specific pathways depending on the metabolic needs. Within this context, we can think about a dietary “metabolic enhancer” as a potential approach within an anti-obesity therapy.

There is also a need to consider the potential pharmacotherapies that might be prescribed at the same time with a specific diet. For instance, a specific diet could activate a pathway or a biochemical reaction that deactivates a pharmacological agent and thus results in therapeutic inefficacy. Therefore, related pharmacovigilance [205] to map the line between the pharmacology and the toxicology [206,207] both in vivo and in vitro [208] remains required. However, the interaction between diet and pharmacology could be positive as shown by the omega-3 polyunsaturated fatty acid that improve the responsiveness to ursodeoxycholic acid during autoimmune liver diseases and cholestatic [209]. Thus, a specific diet choice could turn into a therapeutic adjuvant.

The education and the culture of the lifestyle habits related to diet should not be limited to the caloric balance and nutritional needs, such as vitamins and minerals, but should go beyond that toward the concept of “nutraceutical” [87,210] as a pharmacologically active food with potential therapeutic applications. Such approaches will provide additional tools to manage diseases, such as obesity, ageing and metabolic disorders and thus, optimize medical care.

## Data Availability

Not applicable.
